# Ultra-small superparamagnetic iron oxide nanoparticles for intra-articular targeting of cartilage in early osteoarthritis

**DOI:** 10.1093/rb/rbad052

**Published:** 2023-05-11

**Authors:** Jun Wu, Changqiang Wu, Zhongyuan Cai, Haojie Gu, Li Liu, Chunchao Xia, Su Lui, Qiyong Gong, Bin Song, Hua Ai

**Affiliations:** Institute for Disaster Management and Reconstruction, Sichuan University, Chengdu 610207, China; Medical Imaging Key Laboratory of Sichuan Province, School of Medical Imaging, North Sichuan Medical College, Nanchong 637000, China; Medical Imaging Key Laboratory of Sichuan Province, School of Medical Imaging, North Sichuan Medical College, Nanchong 637000, China; National Engineering Research Center for Biomaterials, Sichuan University, Chengdu 610064, China; National Engineering Research Center for Biomaterials, Sichuan University, Chengdu 610064, China; National Engineering Research Center for Biomaterials, Sichuan University, Chengdu 610064, China; Department of Radiology, West China Hospital, Sichuan University, Chengdu 610041, China; Department of Radiology, West China Hospital, Sichuan University, Chengdu 610041, China; Department of Radiology, Huaxi MR Research Center (HMRRC), Frontiers Science Center for Disease-Related Molecular Network, State Key Laboratory of Biotherapy, West China Hospital, Sichuan University, Chengdu 610041, China; Functional and Molecular Imaging Key Laboratory of Sichuan Province, Key Laboratory of Transplant Engineering and Immunology, NHC, Research Unit of Psychoradiology, Chinese Academy of Medical Sciences, Chengdu 610064, China; Department of Radiology, West China Xiamen Hospital of Sichuan University, Fujian, Xiamen 361000, China; Department of Radiology, West China Hospital, Sichuan University, Chengdu 610041, China; Department of Radiology, Sanya People’s Hospital, Hainan, Sanya 572000, China; National Engineering Research Center for Biomaterials, Sichuan University, Chengdu 610064, China; Department of Radiology, West China Hospital, Sichuan University, Chengdu 610041, China

**Keywords:** early osteoarthritis, magnetic resonance imaging, superparamagnetic iron oxide, collagen type II, targeting peptide

## Abstract

Early diagnosis of osteoarthritis (OA) is critical for effective cartilage repair. However, lack of blood vessels in articular cartilage poses a barrier to contrast agent delivery and subsequent diagnostic imaging. To address this challenge, we proposed to develop ultra-small superparamagnetic iron oxide nanoparticles (SPIONs, 4 nm) that can penetrate into the matrix of articular cartilage, and further modified with the peptide ligand WYRGRL (particle size, 5.9 nm), which allows SPIONs to bind to type II collagen in the cartilage matrix and increase the retention of probes. Type II collagen in the cartilage matrix is gradually lost with the progression of OA, consequently, the binding of peptide-modified ultra-small SPIONs to type II collagen in the OA cartilage matrix is less, thus presenting different magnetic resonance (MR) signals in OA group from the normal ones. By introducing the AND logical operation, damaged cartilage can be differentiated from the surrounding normal tissue on T1 and T2 AND logical map of MR images, and this was also verified in histology studies. Overall, this work provides an effective strategy for delivering nanosized imaging agents to articular cartilage, which could potentially be used to diagnosis joint-related diseases such as osteoarthritis.

## Graphical abstract

**Figure:**
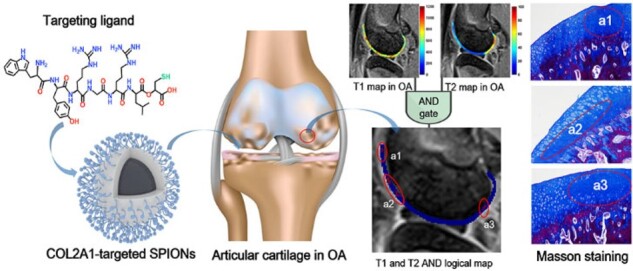

## Introduction

As a degenerative disease characterized by cartilage damage with chronic pain and functional impairment, osteoarthritis (OA) not only reduces the quality of life of patients, but also leads to a growing social health care burden [1, 2]. Although painkiller and artificial joint replacement are available for symptomatic relief of advanced OA, therapies to cure the disease radically are still lacking [3, 4]. Therefore, early diagnosis and intervention of OA is particularly important to slow down the progression of the disease, improve quality of life in patients with OA.

Medical imaging is an important method to assess the structure of cartilage visually. Compared to computed tomography (CT) with radiation of X-ray, magnetic resonance imaging (MRI) has the advantages of non-radiation and high soft tissue resolution, which is suitable for periodic assessment of disease progression and has been widely employed to evaluate cartilage degeneration. However, for early lesions of OA, the sensitivity of MRI itself is not enough to make the diagnosis accurately, and contrast agents are required to improve the sensitivity [2, 5–7].

Articular cartilage, an avascular connective tissue with a joint cavity wall surrounded by synovial membrane, makes it difficult for drugs or contrast agents to enter the articular cartilage through the blood vessels. Consequently, intra-articular injection appears to be a promising method. However, the low-molecular contrast agents or drugs injected into the joint cavity will be rapidly cleared by transmembrane penetration and lymphatic drainage within several hours [8–11]. The non-specific distribution of small molecule contrast agents with insufficient relaxivity leads to low sensitivity in the early diagnosis of OA.

The nanoplatform provides opportunity for improved relaxivity of contrast agents, enhancement of specific distribution, and maintenance of effective concentration [3, 4]. Size of nanoparticles is a principal factor for the ability to pass through gliding surface and enter the articular cartilage, for superficial zone do not covered by perichondrium but is a structure formed by dense collagen fibers arranged in parallel, the gaps of which is around 60 nm [12]. In addition, the extracellular matrix (ECM) of cartilage is rich in proteoglycans, which consists of a large number of branched side chains (glycosaminoglycans) with the gaps of about 20 nm [13]. Therefore, ultra-small nanoparticles (<10 nm) are promising candidates for diagnostic and visual delivery of drugs targeting articular cartilage defects in the knee.

**Scheme 1.**
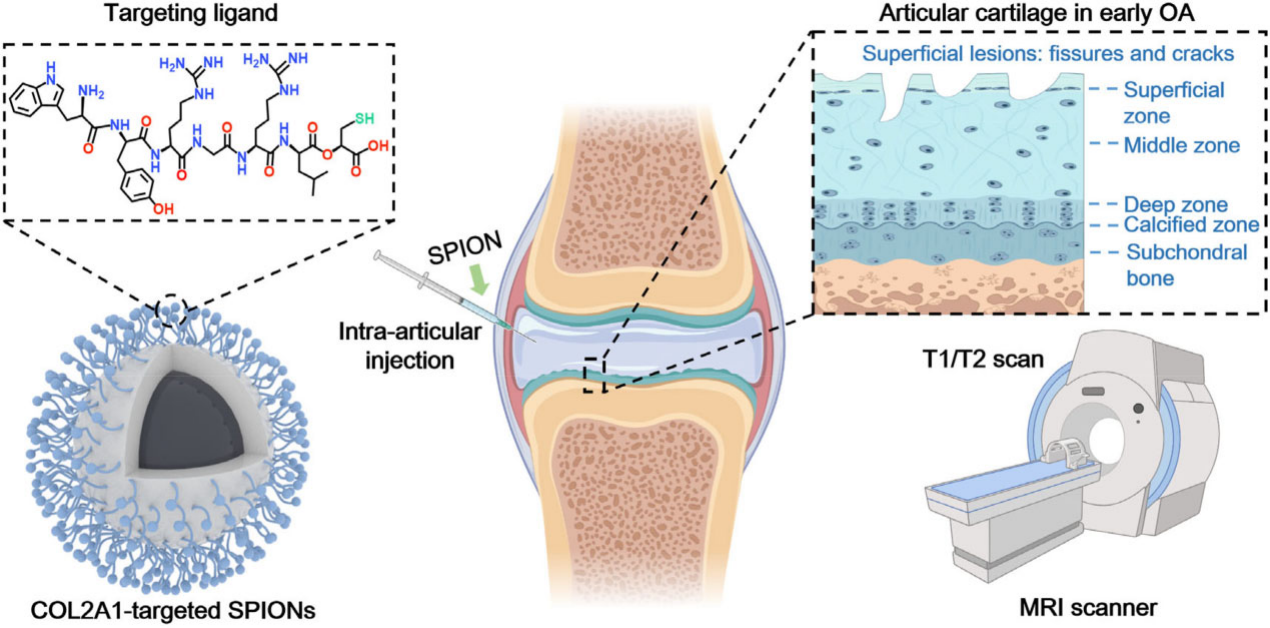
Schematic diagram of COL2A1-targeted SPIONs for articular cartilage in early OA diagnosis by enhanced MR imaging.

Superparamagnetic iron oxide nanoparticles (SPIONs) with good biocompatibility and considerable relaxivity have been widely used in MRI contrast agent studies [14, 15]. In aqueous solution, well-dispersed ultra-small SPIONs exhibit good T1 relaxation performance and can be used in T1-enhanced MRI [16, 17]. Therefore, the synthesis of SPIO crystals with excellent magnetic properties and the controllable dispersion are the focus of research in the design and development of SPIONs [18, 19]. Herein, ultra-small SPIONs were synthesized by organic high-temperature thermal decomposition method, and then transferred into the aqueous phase with the assistant of PEG-coating. The as-synthesized SPIO (SPIO@PEG) with a particle size of 5.9 ± 1.1 nm in aqueous phase is suitable for penetrating into cartilage. Furthermore, to be effectively retained in the extracellular matrix of cartilage, surface modification was performed by linking **WYRGRL**, a short peptide targeting collagen type II α1 chain (COL2A1) existing in the cartilage [20, 21]. The resulting contrast agent (SPIO@PEG-peptide) not only has active targeting capabilities to cartilage, also can be used as a dual-mode T1/T2 MRI probe due to its significant T1 reinforcement and also suitable T2 enhancement caused by moderate particle size [19]. The well-established rabbit model of osteoarthritis was used to test if the contrast agent was able to differentiate normal cartilage from early OA (Scheme 1).

## Materials and methods

### Materials

Acetylacetonate iron (99.9%), oleic acid (90%), oleylamine (70%), 1,2-hexadecanediol (97%), diphenyl ether (99%), monomethyl polyethylene glycol (mPEG-OH, molecular weight (Mw): 550 Da), and dopamine, were purchased from Sigma-Aldrich LLC. (St. Louis, MO, USA). Maleimide polyethylene glycol succinimidyl carboxymethyl ester (MAL-PEG2000-SCM) was purchased from Beijing Jenkem Technology Co., Ltd. (Beijing, China). Triethylamine was purchased from Beijing KeyKem Technology Co., Ltd. (Beijing, China). Tetrahydrofuran, acetone, n-hexane, anhydrous ethanol and other organic solvents were purchased from Chengdu Kelong Chemical Reagent Factory (Chengdu, China) and used directly. Peptide WYRGRL targeting cartilage matrix component collagen type II α1 was provided by ChinaPeptides Co. Ltd (Shanghai, China), which has been acetylated with a cysteine at the carboxy (C) terminus: Ac-WYRGRLC(Mw: 995.13, Purity: 97.43%, Supplementary Fig. S1). Papain: Molecular weight 23 406 Da, 10 units/mg, lyophilized powder, CAS number 9001-73-4, was from Sigma-Aldrich LLC. (St. Louis, MO, USA). Six-month-old New Zealand white rabbits with a body weight of 3.0–3.5 kg were purchased from Sichuan Dossy Experimental Animal Co., Ltd. (Chengdu, China).

### Characterization

Transmission electron microscopy (TEM) images were obtained on (FEI Tecnai 20 microscope, Netherlands). ^1^H NMR spectra were obtained in a Bruker Avance III HD 400 MHz NMR spectrometer (Bruker Inc., Switzerland). The size and zeta potential of nanoparticles were obtained on a Malvern Zeta sizer system (Zeta sizer Nano ZS, Malvern Instruments, Ltd, UK). MR imaging were performed on clinical MR scanners (3.0 T MAGNETOM Skyra, Siemens, Germany and 1.5 T Siemens Sonata, Siemens, Germany).

### Synthesis of COL2A1-targeted ultra-small SPIONs

**Figure 1.**
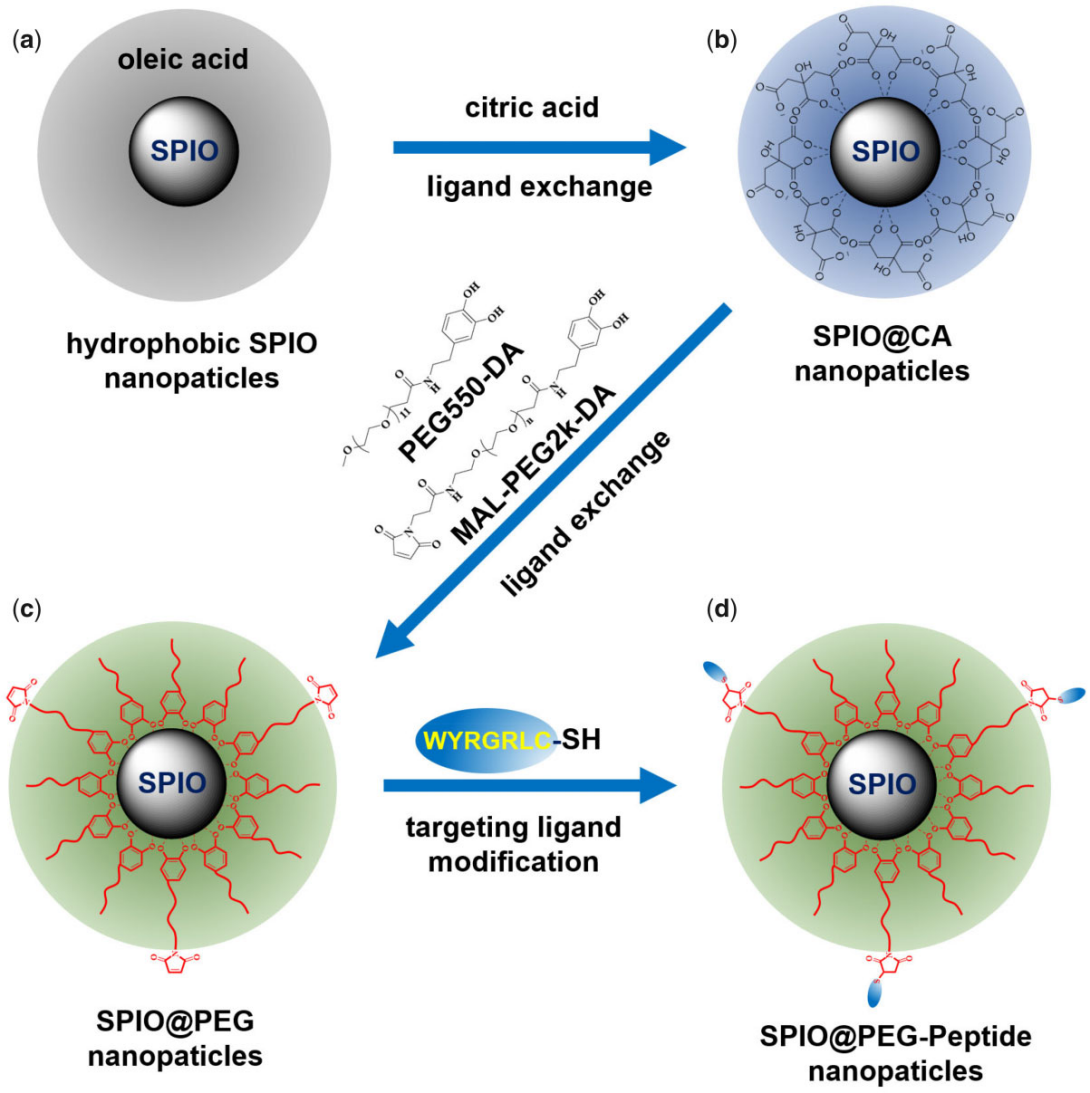
The preparation route of the collagen type II α1 chain-targeted SPIO@PEG-Peptide. Abbreviations: CA, citric acid; DA, dopamine.

As shown in Fig. 1, the preparation of COL2A1-targeted Ultra-small SPIONs consists of four steps. Firstly, according to previously reported methods, oleic acid-coated ultra-small hydrophobic SPIO nanocrystals were synthesized by high-temperature thermal decomposition in organic phase [21–24]. Secondly, water-dispersed ultra-small iron oxide nanoparticles (SPIO@CA) were obtained by ligand exchange with citric acid as the coating. Then, mPEG-dopamine and maleimide-PEG-dopamine was decorated onto the surface of SPIO surface by ligand exchange. Finally, the maleimide group on the surface of SPIO@PEG reacted with WYRGRLC-SH to obtain COL2A1-targeted Ultra-small SPIONs. The detailed synthesis steps are as the following:

### Synthesis of water-dispersed ultra-small superparamagnetic iron oxide nanoparticles (SPIO@CA)

Oleic acid-coated ultra-small hydrophobic SPIO with about 4 nm nanocrystals were exchanged with citric acid to obtain water-dispersed SPIO@CA. Briefly, 1 g of citric acid was dissolved in 70 ml of deionized water. After the solution was adjusted to pH 7.0 with NaOH, 110 ml acetone, 70 ml hexane and 60 mg of oleic acid-coated SPIO were added in turn. The mixture was stirred and heated to 75°C for 48 h under inert gas. After the reaction, the solution was cooled to room temperature and centrifuged at 6000 rpm/min for 10 min. The black precipitates were collected and dissolved in 10 ml deionized water, and then filtered by microporous filter membrane to obtain water-dispersed SPIO@CA (Fig. 1b).

### Synthesis of mPEG-dopamine (PEG0.55k-DA)

Five hundred fifty milligrams of mPEG-OH (1 mmol, Mw: 550 Da) were added into a round-bottom flask and dried under vacuum for 2 h. Twenty milliliters of dried THF were added to dissolve under inert gas. Under magnetic stirring, 320 mg of N, N′-carbonyl diimidazole (CDI) was added and heated to reflux for 12 h. After cooling to room temperature, 10 µl deionized water was added and stirred for 5 minutes. Then, solvent was removed by rotary evaporation and dried under vacuum for 2 h. Fifty milliliters of THF were added to dissolve the residue, and 200 mg triethylamine and 380 mg dopamine hydrochloride (2 mmol) were added in. The mixture was stirred at room temperature for 24 h. After the reaction, the solvent was removed by rotary evaporation. The product was dissolved in 50 ml methylene chloride, washed three times with saturated salt solution. The solvent was removed and terminal dopamine-modified polyethylene glycol (mPEG-DA) was obtained.

### Synthesis of maleimide-PEG-dopamine (MAL-PEG2k-DA)

MAL-PEG2000-SCM (1 g) was weighed to a round-bottom flask, 50 ml THF was added under inert gas. Then, 60 mg triethylamine and 114 mg dopamine hydrochloride (0.6 mmol) were added in. The mixture was stirred at room temperature for 24 h. After the reaction, the solvent was removed by rotary evaporation and residue was dissolved in 50 ml methylene chloride, washed three times with saturated salt solution. The solvent was removed to obtain MAL-PEG2k-DA.

### Preparation of PEG-coated SPIONs (SPIO@PEG)

mPEG-DA (20 mg) was dissolved in 1 ml deionized water and mixed with 2 ml hydrophilic ultra-small SPIO nanocrystals solution (Fe: 4 mg/ml) at room temperature. The mixture was heated to 50°C for 48 h under inert gas. Then, the solution was dialyzed with deionized water for 2 days to obtain PEG-coated SPIONs (SPIO@PEG)

Maleimide modified SPIO@PEG nanoparticles were prepared with the same process, except that 20 mg mPEG-DA was replaced by 15 mg mPEG-DA and 5 mg MAL-PEG2k-DA (Fig. 1c).

### Short peptide modification of SPIO@PEG nanoparticles

Sulfhydryl modified short peptides (1 mg, Ac-WYRGRLC-SH) were dissolved in 200 μl DMSO, then added into maleimide modified SPIO@PEG nanoparticle solution, and reacted overnight at room temperature. The solution was dialyzed for 2 days to obtain short peptide-modified SPIO@PEG nanoparticles (SPIO@PEG-Peptide, Fig. 1d).

### Relaxivity measurement and phantom of SPIO@ PEG nanocrystals *in vitro*

The relaxivity (*r*) is used to describe the ability of contrast agents to change longitudinal relaxation time (T1) or transverse relaxation time (T2), and which is typically defined as the slope of the equation (1). It represents the linear relationship between different concentrations of contrast agents (CA) and the corresponding relaxation rate (i.e. the reciprocal of the relaxation time, 1/T1 or 1/T2) [25]. Its functional relationship can be described by the following formula: where *r*_1_ and *r*_2_ represent the T1 and T2 relaxivity of the contrast agent, respectively. The ${\left(\frac{1}{T_{1}}\right)}_{\mathrm{obs}}$ and ${\left(\frac{1}{T_{2}}\right)}_{\mathrm{obs}}$denotes the observed relaxation rate measured at a certain concentration of the contrast agent, while ${\left(\frac{1}{T_{1}}\right)}_{d}$ and ${\left(\frac{1}{T_{2}}\right)}_{d}$ represents the relaxation rate of the (diamagnetic) solvent or tissue without any extra contrast agent, which can be considered as a constant [26].


(1) 1T1obs=1T1d+r1×[CA]1T2obs=1T2d+r2×[CA]

The T1-weighted and T2-weighted images (T1WIs and T2WIs) of phantoms of SPIO@PEG in water were obtained using the clinical 1.5 T and 3.0 T MR Scanners. T1WI imaging protocol utilized spin echo (SE) sequences with a range of repetition times (TR) including 20, 100, 300, 500, 700, 900, 1100, 1500, 1900, 2300 and 3100 ms, and echo time (TE)=5.3 ms, in which a total of 11 data points were used for non-linear fitting to calculate the longitudinal relaxation time. And meanwhile, T2 WIs also employed the SE sequences with TR = 5000 ms, and TE = 7, 12, 20, 30, 40, 55 ms, in which 5 data points were measured and analyzed using linear fitting methods to determine the transverse relaxation time.

### Establishment of papain-induced osteoarthritis rabbit model

All the animal experiments were approved by the Experimental Animal Research Committee of Sichuan University. The procedures followed during the experiments were in accordance with the ethical guidelines set by the Experimental Animal Research Committee of Sichuan University, and ensuring that the research is justified based on scientific and ethical considerations.

Papain enzyme (6 mg, 60 units) was weighed out at one time, and dissolved in saline, resulting in a 3 ml papain enzyme solution with a concentration of 20 units/ml and used within 1 h. The dosage of injection was 5 units/leg (0.25 ml/leg) via intra-articular injection. Taking a half-hour walk daily was adopted for promoting success in osteoarthritis modeling to provide sufficient compressive movement for the cartilage and help the interaction between papain and cartilage.

### MRI of articular cartilage *in vivo*

Both the two types of contrast agents (SPIO@PEG and SPIO@PEG-Peptide) mentioned above had been diluted to a concentration of 0.5 mg/ml in saline, and a dosage of 0.25 ml/leg (125 μg/leg)was injected each time.

All rabbits were kept under anesthesia with 2.0% isoflurane in oxygen for the duration of the MRI experiment and were not given ventilation support, which allowed them to breathe spontaneously. In this experiment, 6 rabbits were divided into two groups randomly, with 3 rabbits in each group. The first group did not undergo modeling but only received injections of contrast agent as the normal group. The second group was OA group, which underwent modeling and also received injections of contrast agent. In both groups, the left legs were injected with the SPIO@PEG-Peptide and the SPIO@PEG was injected into the right one via intra-articular injection. The same batch of contrast agent was used for both the normal group and the OA group.

The retention of the contrast agent in normal and OA articular cartilage is then analyzed through magnetic resonance quantitative imaging *in vivo*. T1 WIs and T2 WIs of articular cartilage were acquired on both the left and right legs of each rabbit, at the time before injection, 24, 48 and 96 h post-injection, with a scanning time of 40 minutes per leg on clinical 3.0 T MRI using SE sequences. The sequence parameters of T1 WIs were TE = 12 ms, TR = 800, 1000, and 1200 ms, while T2 WIs were acquired with TR = 2800 ms, TE = 13, 25, 38, 50 and 63 ms, and both sequences were with a slice thickness of 1 mm and FOV matrix resolution (pixels) of 512 × 512. The scanning process is designed to ensure that both the joint position and pixel positions of the corresponding cartilage T1 and T2 weighted images remains unchanged, in order to be applied to subsequent T1 map and T2 map logical gate AND operations.

### Logical and operation

Here, logical AND operation that can eliminate false errors in T1 and T2 weighted images was demonstrated in Supplementary Fig. S2. Ultra-small SPIONs as dual-mode contrast agents were used to perform T1-weighted imaging and T2-weighted imaging simultaneously on the same position of tissues. These high quality images provides an opportunity to generate a fused image of T1 mapping and T2 mapping of articular cartilage in OA to better differentiate lesion areas by using the ‘AND logic gate’ process [27, 28].

### Statistical analysis

The T1 and T2 of articular cartilages in the normal and OA group with and without SPIONs (targeted and non-targeted) were determined using MRI *in vivo*. The statistical analysis was performed using ANOVA in IBM SPSS V27. A *P*-value of less than 0.05 was considered statistically significant compared to the control.

## Results and discussion

### Synthesis of mPEG-DA and MAL-PEG-DA

mPEG-DA molecules were synthesized by first activating mPEG550-OH using CDI, to produce an intermediate mPEG 550-CI, and then reacted with dopamine. The dopamine-modification of PEG is verified by ^1^HNMR spectroscopy of mPEG550-OH, mPEG550-CI and mPEG550-DA. The appearance of characteristic peaks of the intermediate mPEG 550-CI and the final product mPEG 550-DA indicated the successful attachment of dopamine at the end of PEG (Supplementary Fig. S3).

On the other hand, the synthesis of MAL-PEG-DA involves using a directly purchased two-end functionalized MAL-PEG2k-SCM, which contains both maleimide and SCM functional groups. This reagent is then reacted with dopamine to produce dopamine and maleimide functionalized MAL-PEG-DA molecules. The reactant and product were then verified by ^1^HNMR spectroscopy, and the corresponding spectra and characteristic peaks were obtained (Supplementary Fig. S4). These results indicate the successful synthesis of MAL-PEG-DA.

### Synthesis of PEG@SPIO nanocrystals

**Figure 2.**
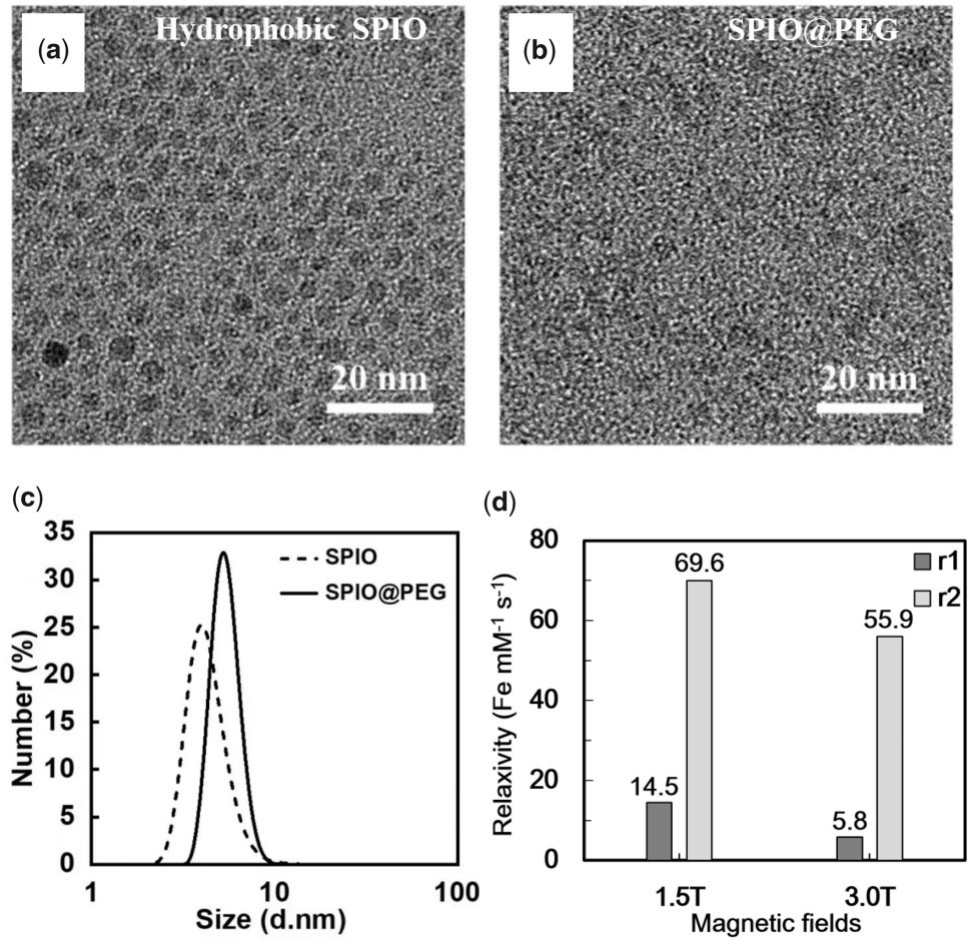
TEM of hydrophobic SPIO nanocrystal particles with oleic acid coating (**a**) and hydrophilia SPIO@PEG nanoparticles (**b**). DLS distribution of hydrophobic SPIO nanoparticles (4.4 ± 1.1 nm) and hydrophilia SPIO@PEG nanoparticles (5.9 ± 1.1 nm) (**c**). the longitudinal relaxivity (*r*_1_) and transverse relaxivity (*r*_2_) of ultra-small SPIO@PEG nanoparticles at 1.5 T and 3.0 T (**d**).

In this study, hydrophobic SPIO nanocrystals with oleic acid coating were first prepared. Water-dispersed SPIO@CA were obtained by ligand exchange with citric acid as the coating material. Then, PEG-DA was added and exchanged in aqueous solution to prepare PEG coated SPIONs, SPIO@PEG. The size distribution and dispersion of the crystal particles were analyzed by DLS and TEM. As shown in Fig. 2a and b, the hydrophobic SPIO and hydrophilic SPIO@PEG nanoparticles were still single crystal dispersed nanoparticles. DLS results show that they have narrow size distribution, with an average particle size of 4.4 ± 1.1 and 5.9 ± 1.1 nm, respectively (Fig. 2c), and the zeta potential of SPIO@PEG was measured to be −7.3 ± 2.6 mV.

### Relaxivity measurement of SPIO@PEG *in vitro*

To evaluate the ability of contrast agent to shorten relaxation time of the tissues, longitudinal relaxivity (*r*_1_) and transverse relaxivity (*r*_2_) of SPIO@PEG were measured in clinical MRI scanners (Supplementary Figs S5 and S6).The results indicate that they have noteworthy longitudinal relaxivity with *r*_1_ values of 14.5 Fe mM^−1^s^−1^ and 5.8 Fe mM^−1^s^−1^ under 1.5 T and 3.0 T MRI respectively (Fig. 2d), significantly higher than that of the commercial T1 contrast agent Gd-DTPA (Magnevist™) with a *r*_1_ value of 4.95 Gd mM^−1^s^−1^. Furthermore, the ultra-small SPIO@PEG also have proper transverse relaxivity with *r*_2_ values of 69.6 Fe mM^−1^s^−1^and 55.9 Fe mM^−1^s^−1^ under 1.5 T and 3.0 T MRI respectively (Fig. 2d), which are slightly lower than commercial T2 contrast agents based on SPIO (Feraheme™) with a *r*_2_ value of 89 Fe mM^−1^s^−1^ under 1.5 T. According to literature reports, a lower *r*_2_ value is beneficial for positive (T1) contrast agents [19]. Monodisperse ultra-small SPIONs have an intermediate *r*_2_/*r*_1_ ratio, and can be used for contrast enhancement in T1–T2 dual-mode MRI. The results showed that the ultra-small SPIONs with relatively balanced T1 and T2 relaxivities (*r*_2_/*r*_1_ = 9.6) under 3.0 T, is an ideal T1–T2 dual-mode contrast agent [24].

### Pathological analysis of articular cartilage

All the six rabbits of normal group and OA group, were sacrificed by injecting air via ear vein with deep respiratory anesthesia after 96 h of MR imaging. Then, the femoral condyles were removed and stained with H&E, Safranin O and Masson to evaluate the overall cartilage, chondrocyte, extracellular matrix, proteoglycan and type II collagen (Supplementary Fig. S7). Safranin O staining revealed changes in proteoglycan content in the extracellular matrix [29, 30]. It was found that proteoglycan content decreased with the progression of OA, compared with the normal group. Masson staining showed that the collagen fibers on the superficial zone of articular cartilage in the OA group were significantly reduced (Supplementary Fig. S7d). H&E staining revealed some lesions such as fissures and cracks occurred on the superficial zone of cartilage in OA group, by contrast, the cartilage surface was intact and smooth in normal group. These results indicate the successful establishment of the early OA rabbit models induced by papain.

### T1 mapping and T2 mapping of *in vivo* MRI

**Figure 3.**
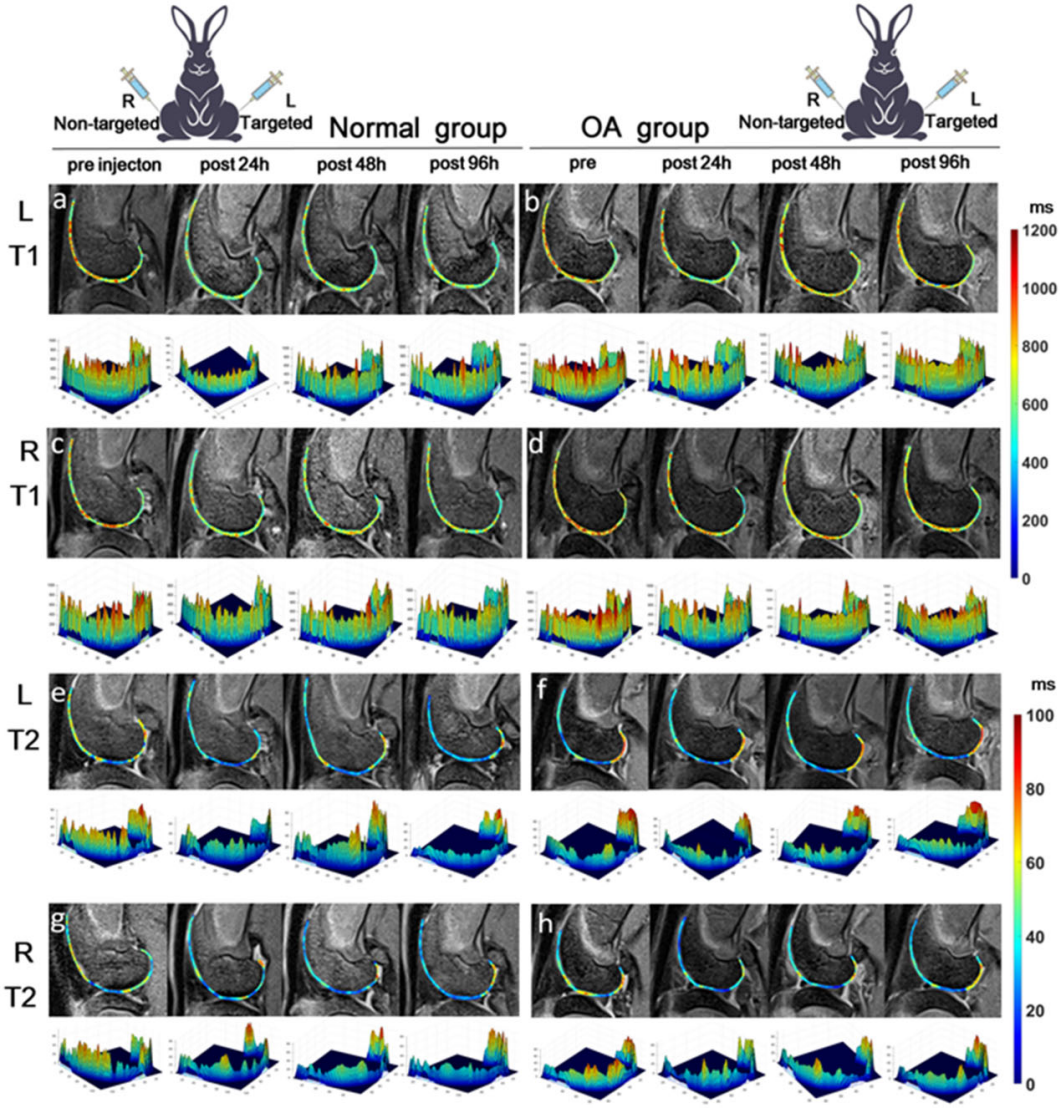
T1 map and T2 map of articular cartilage MR imaging *in vivo*. T1 heat map (**a–d**) and T2 heat map (**e–h**) of ROI at pre, 24, 48 and 96 h post-injection of the targeted SPIO@PEG-peptide (left leg, a, b, e, f) and non-targeted SPIO@PEG (right leg, c, d, g, h) in normal and OA group, and the corresponding 3D curve of ROI.

**Figure 4.**
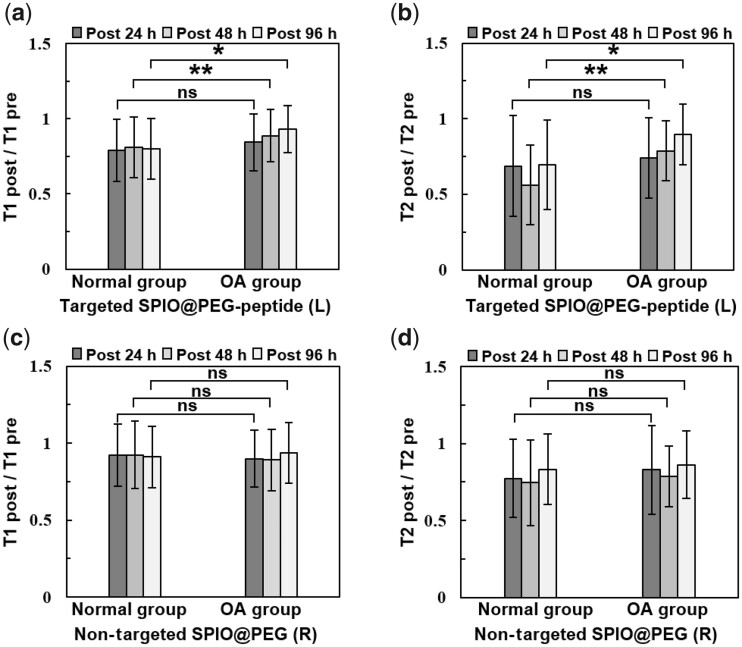
The ratio of relaxation time (T1 post/T1 pre and T2 post/T2 pre) at post 24, 48 and 96 h after injection of the targeted SPIO@PEG-peptide (**a, b**) and non-targeted SPIO@PEG (**c, d**) in normal and OA group (*n* = 6). (**P < 0.01 and *P < 0.05).

There are specific methods for determining T1 and T2 relaxation times of articular cartilage using Matlab 2013 with the algorithm shown in Supplementary Algorithm S1. The two-point method can be used to determine T1 relaxation time, while the linear fitting method can be used for T2 relaxation time [31]. As shown in Figs 3 and 4, the T1 and T2 relaxation time of the cartilage has significantly decreased after the injection of the contrast agent in both normal group and OA group. The T1, T2 and ratio of T1 post/pre and T2 post/pre values of the ROI were used to quantify the effect of contrast agents in each group (Supplementary Table S1).

It can be observed that the T1 values of the normal group in Fig. 3a and c are significantly lower in post 48 and 96 h by comparison with those of the OA group in Fig. 3b and d, as indicated by the bluish color in the heat map. Normalizing the T1 values at each time point after the injection of contrast agent by dividing them by the T1 value before injection of the contrast agent (T1 post/T1 pre) is to eliminate individual differences between samples, so that longitudinal comparisons can be statistically analyzed. The relaxation times of T1 and T2 decreased at 24 h post-injection in both normal or OA groups, no matter injected with SPIO@PEG or SPIO@PEG-peptide (Fig. 4a–h). Noteworthy, with the assistant of COL2A1-targeted WYRGRL, there may be more retention of nanoparticles in the cartilage injecting with SPIO@PEG-peptide than non-targeted SPIO@PEG at 24 h, indicated by the T1 post/pre and T2 post/pre of SPIO@PEG-peptide is lower than that of the SPIO@PEG. For example, the normal group of T1 post/pre of SPIO@PEG-peptide vs that of SPIO@PEG is 0.79 vs 0.92 at 24 h, and that of OA group is 0.84 vs 0.90 in Fig. 4a and c (Supplementary Table S1).

Figure 4a showed that there is a significant difference between the normal group and the OA group at 48 and 96 h after the injection of targeted contrast agent, while there is no statistical difference between the normal group and the OA group injected with non-targeted contrast agent at any time point (Fig. 4c). The results suggest that the degradation of collagen fibers in the articular cartilage caused by papain, leading to the removal of partial COL2A1-attached SPIO@PEG-peptide via cartilage clearance mechanism, which results in the increase of T1 value. The T1 relaxation time may be a useful tool in the evaluation and monitoring the articular cartilage degeneration with assistant of targeted contrast agents SPIO@PEG-peptide enhanced MRI in the OA process [32–35].

T2 relaxation time was reported that it can be used as a biological index to judge the degeneration of OA cartilage [36–39]. It is noteworthy that injection of contrast agents significantly decreased T2 values of the cartilage, indicating that ultra-small SPIONs can be used as a dual-mode T1–T2 contrast agent. Supplementary Table S1 demonstrate that the T2 value changes in the normal and OA groups have a similar trend to the T1 value. Similarly, there is a statistically significant difference between the normal and OA groups at 48 and 96 h after injection of the targeted SPIONs (Fig. 4b), while there is also no statistical difference between these two groups when injected with the non-targeted SPIO@PEG (Fig. 4d).

### Accumulation of SPION in articular cartilage

**Figure 5.**
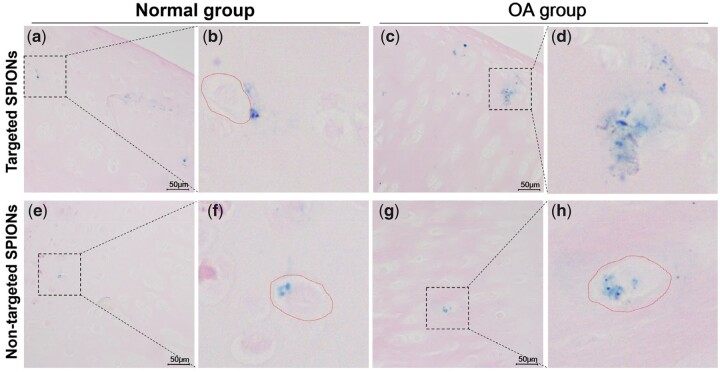
Prussian blue staining of articular cartilage, and red circle denotes chondrocyte. (**a–d**) Left leg with targeted SPIO@PEG-peptide injection. (**e–h**) Right leg with non-targeted SPIO@PEG injection. b, d, f, g are the locally magnification of a, c, e, g by four times, respectively.

Prussian blue staining was used to detect the accumulation of SPIONs in cartilage tissue sections. The results indicated that both targeted SPIO@PEG-peptide and non-targeted SPIO@PEG exhibited significant residual in articular cartilage after injected for 96 h (Fig. 5). Moreover, it was observed that non-targeted SPIO@PEG mainly accumulated in the cavity of chondrocytes (Fig. 5f and h), while targeted SPIO@PEG-peptide had significant deposition in the ECM. This is because that peptide-modified SPIO could evade the ECM clearance through binding to type II collagen, while the non-targeted SPIO@PEG accumulated in the ECM were cleared rapidly. These results suggest that ultra-small SPIONs could penetrate into cartilage tissue, and the phagocytosis of chondrocytes exert a sequestering effect. However, SPIONs accumulated in the ECM would be cleared rapidly, and collagen-targeting peptide modification effectively improves the retention of SPIONs in cartilage tissue.

### And logical operation of T1 and T2 in OA

**Figure 6.**
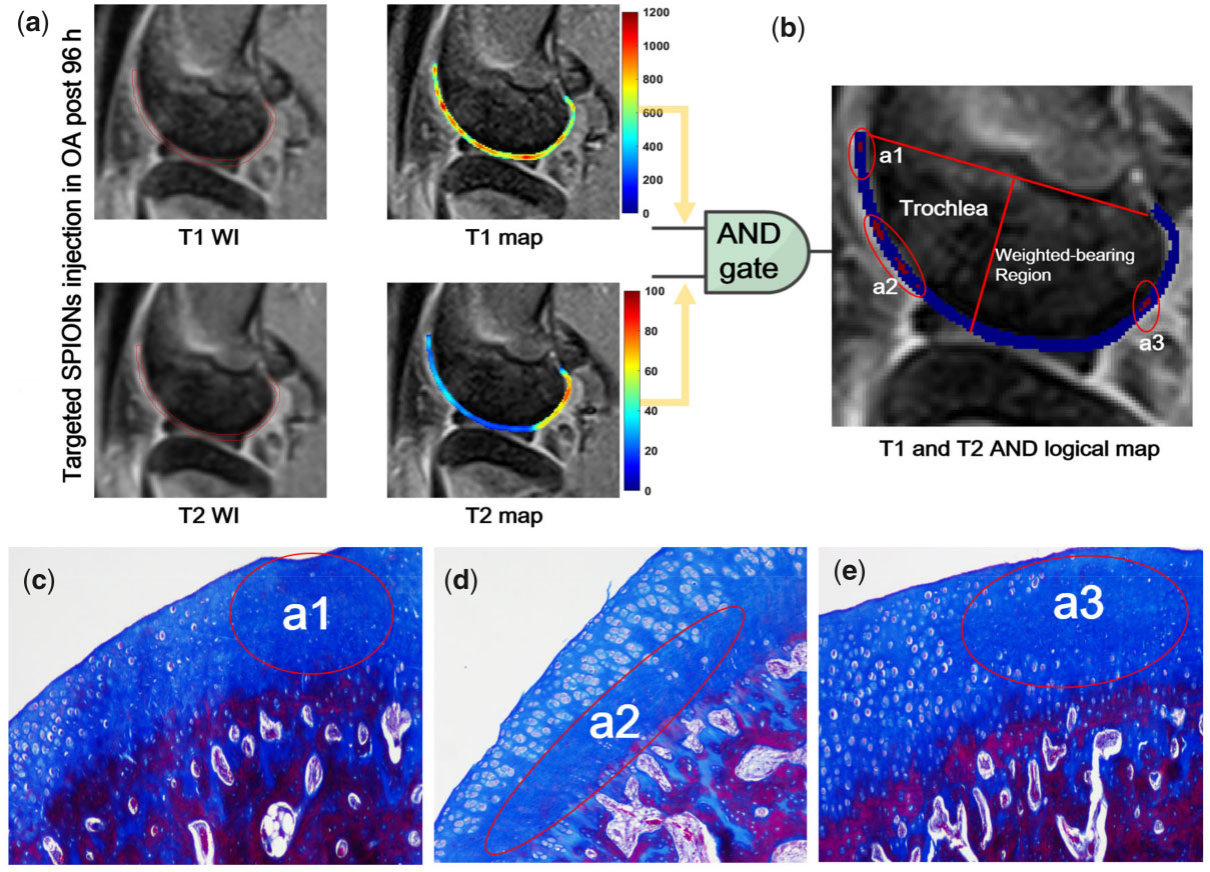
*In vivo* enhanced MRI presenting articular cartilage degeneration in OA using the AND logic operation. (**a**) T1, T2 weighted images and T1, T2 map of ROI. (**b**) AND logic output (red) observed of a1, a2 in trochlea and a3 in weighted-bearing area. (**c–e**) Masson staining corresponding to a1, a2 and a3.

Here, AND logic gate procedure was utilized to filter the interference caused by joint effusion and ligament signals in MRI (Fig. 6).The first step is to manually segment the ROI based on the contour of cartilage in the T1WIs and T2WIs, in which the T1 map and T2 map are then obtained through a fitting algorithm described previously, as shown in Fig. 6a. The next step involves applying the AND logic algorithm to both images, as detailed in Supplementary Algorithm S1. The process results in the highlighting of abnormal regions in red, identified as a1, a2, and a3 in Fig. 6b. These corresponds to the histologically abnormal area as shown in Fig. 6c–e. The a1 and a2 regions appear in the trochlea of the joint femur, near the location of papain injection and contrast agent, while the a3 region appears in the weight-bearing area of the articular cartilage. These damaged and degenerated cartilage regions are also reflected in histology, with a1–a3 corresponding to the abnormal chondrocytes and extracellular matrix area seen in Fig. 6c–e, in which the collagen fiber network structure was broken down by Masson staining. These areas could also be one of the first places to show signs of degeneration and deterioration due to the heavy stress and pressure it is subjected to [40, 41]. As the degeneration progresses, it can lead to the development of joint arthritis and cause pain, stiffness, and reduced mobility in the joint. Early detection and intervention are important to slow down the progression of joint arthritis and preserve the joint’s functionality [42, 43].

## Conclusions

In this work, we presented a design concept of ultra-small SPIONs targeted to the cartilage matrix that can be used as an imaging probe of early OA. Using COL2A1-targeting SPIONs, significant difference in T1 post/pre and T2 post/pre ratios were observed between the OA and the normal animal groups at 48 and 96 h after injection of the probes. By conjugating a bio-affinity ligand WYRGRL to nanoparticle’s surface, limited nanoparticle clearance was found in normal extracellular matrix compared with the damaged ones in the OA group, offering the advantage of differentiating cartilage degeneration in early OA from normal cartilage tissue. By introducing the AND logical operation, one could differentiate damaged cartilage from the surrounding normal tissue on T1 and T2 AND logical map, and also verified in histology. The use of ultra-small nanoparticles as a targeted delivery system in combination with the AND logical operation presents a promising strategy for advancing the diagnosis and treatment of OA.

## Supplementary Material

rbad052_Supplementary_Data
