# Supplementary material for: Ultra-small superparamagnetic iron oxide nanoparticles for intra-articular targeting of cartilage in early osteoarthritis
Source: Regen Biomater. 2023 May 11;10:rbad052. doi: 10.1093/rb/rbad052 (PMC10307945; doi:10.1093/rb/rbad052)
Supplement: rbad052_Supplementary_Data [file rbad052_supplementary_data.docx]

**Supplementary material**

**Ultra-small Superparamagnetic Iron Oxide Nanoparticles for intra-articular targeting of Cartilage in Early Osteoarthritis**

Jun Wu ^1, 3^, Changqiang Wu ^3*^, Zhongyuan Cai ^2^, Haojie Gu ^2^, Li Liu ^2^, Chunchao Xia^4^, Su, Lui^4^, Qiyong Gong^5, 6^, Bin Song ^4^, Hua Ai ^2, 4, *^

1. Institute for Disaster Managenent and Reconstruction, Sichuan University, Chengdu, 610207, P. R. China
2. National Engineering Research Center for Biomaterials, Sichuan University, Chengdu 610064, China
3. Medical Imaging Key Laboratory of Sichuan Province, School of Medical Imaging, North Sichuan Medical College, Nanchong, 637000, P. R. China
4. Department of Radiology, West China Hospital, Sichuan University, Chengdu 610041, China
5. Huaxi MR Research Center (HMRRC), Department of Radiology, West China Hospital of Sichuan University, Chengdu 610041, China
6. Psychoradiology Research Unit of Chinese Academy of Medical Sciences, Sichuan University, Chengdu 610064, China

* Corresponding author:

Hua Ai, National Engineering Research Center for Biomaterials, Sichuan University, Chengdu 610064, P. R. China. Phone: 86-28-85413991, Email: huaai@scu.edu.cn

Changqiang Wu, Medical Imaging Key Laboratory of Sichuan Province, School of Medical Imaging, Affiliated Hospital of North Sichuan Medical College, Nanchong 637000, China. E-mail: wucq1984@nsmc.edu.cn


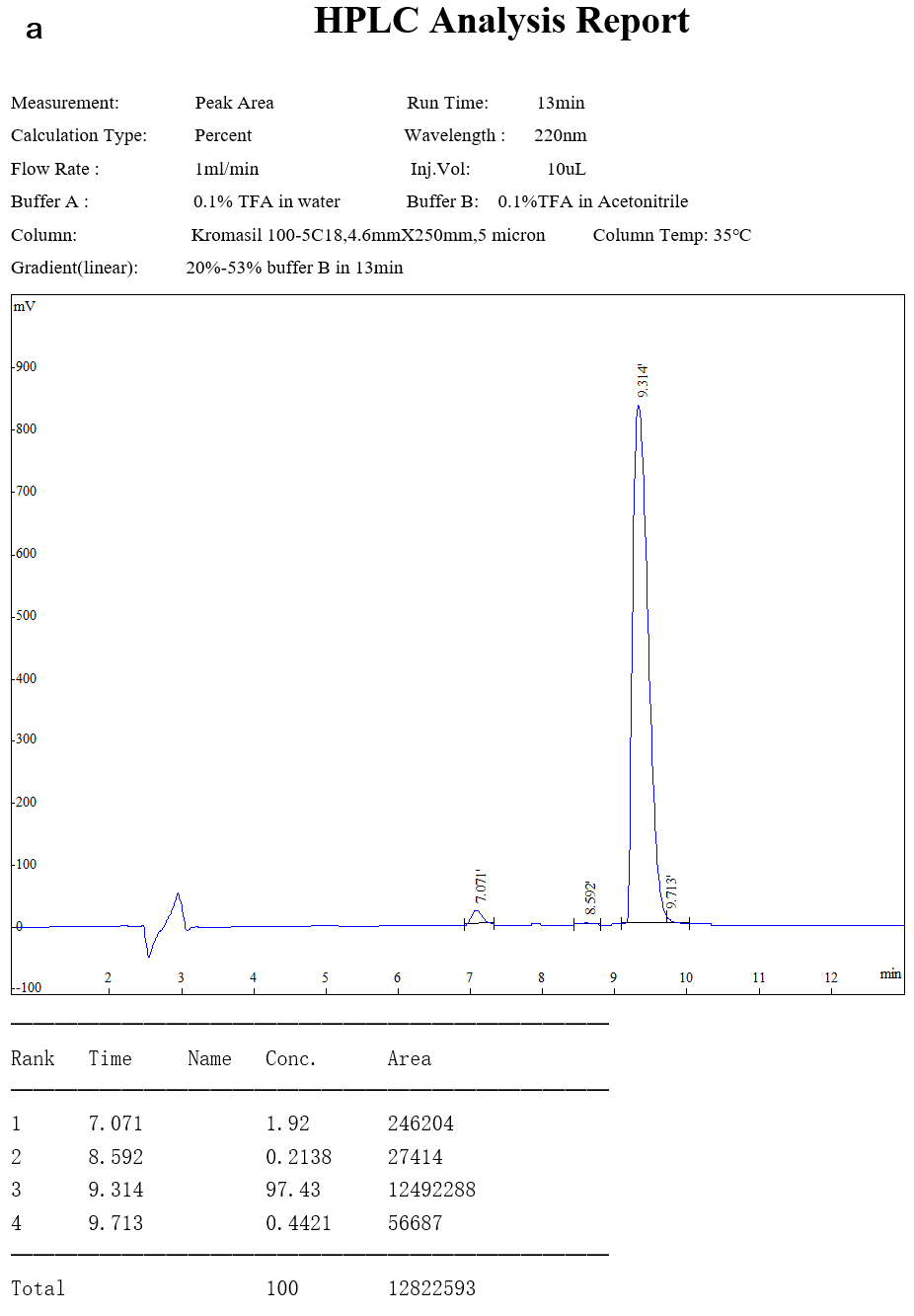

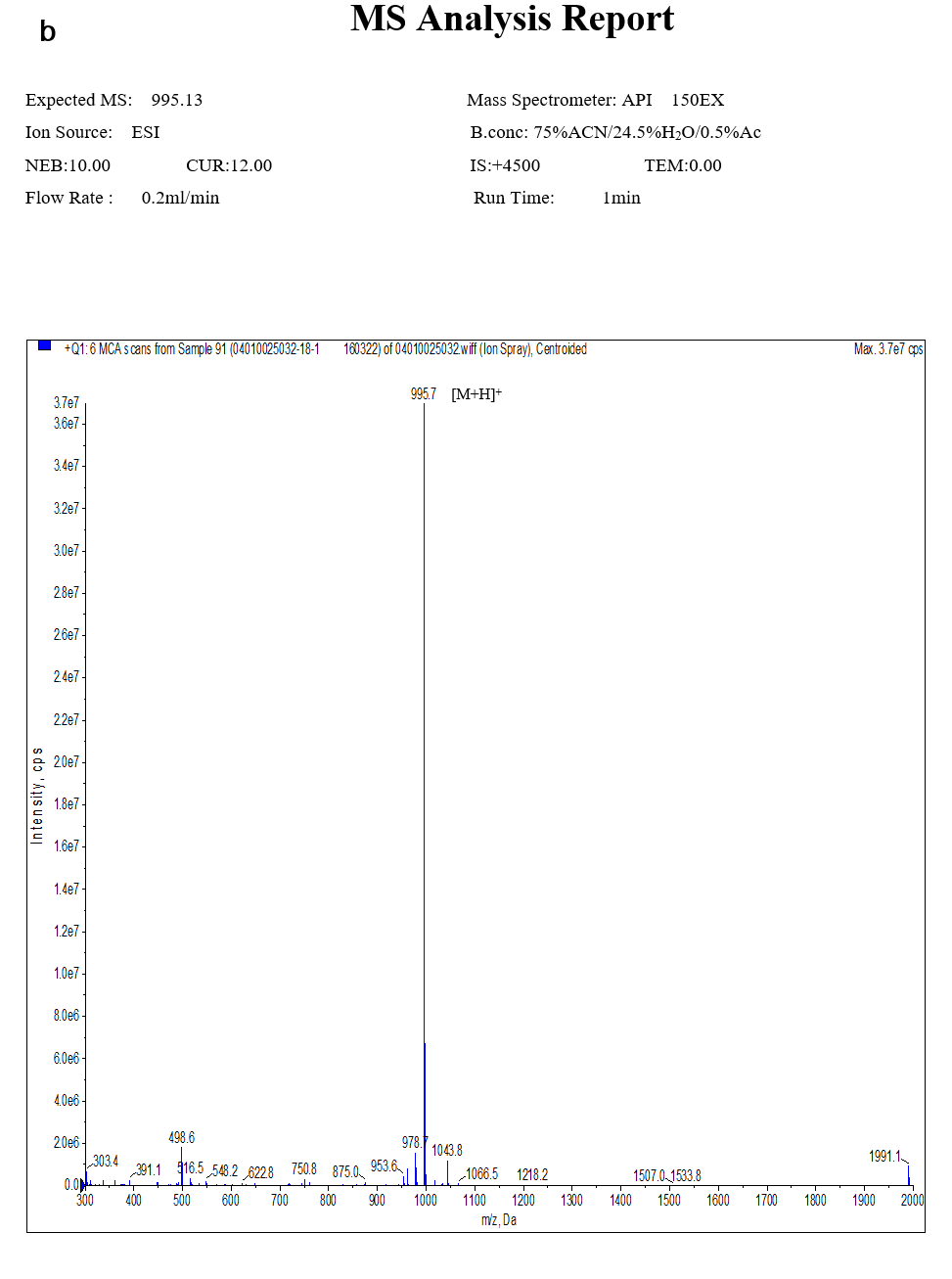


**Figure S1.** The HPLC analysis report (a) and MS analysis report (b) of the peptide Ac-WYRGRLC (Ac, acetylation at the N-terminus).


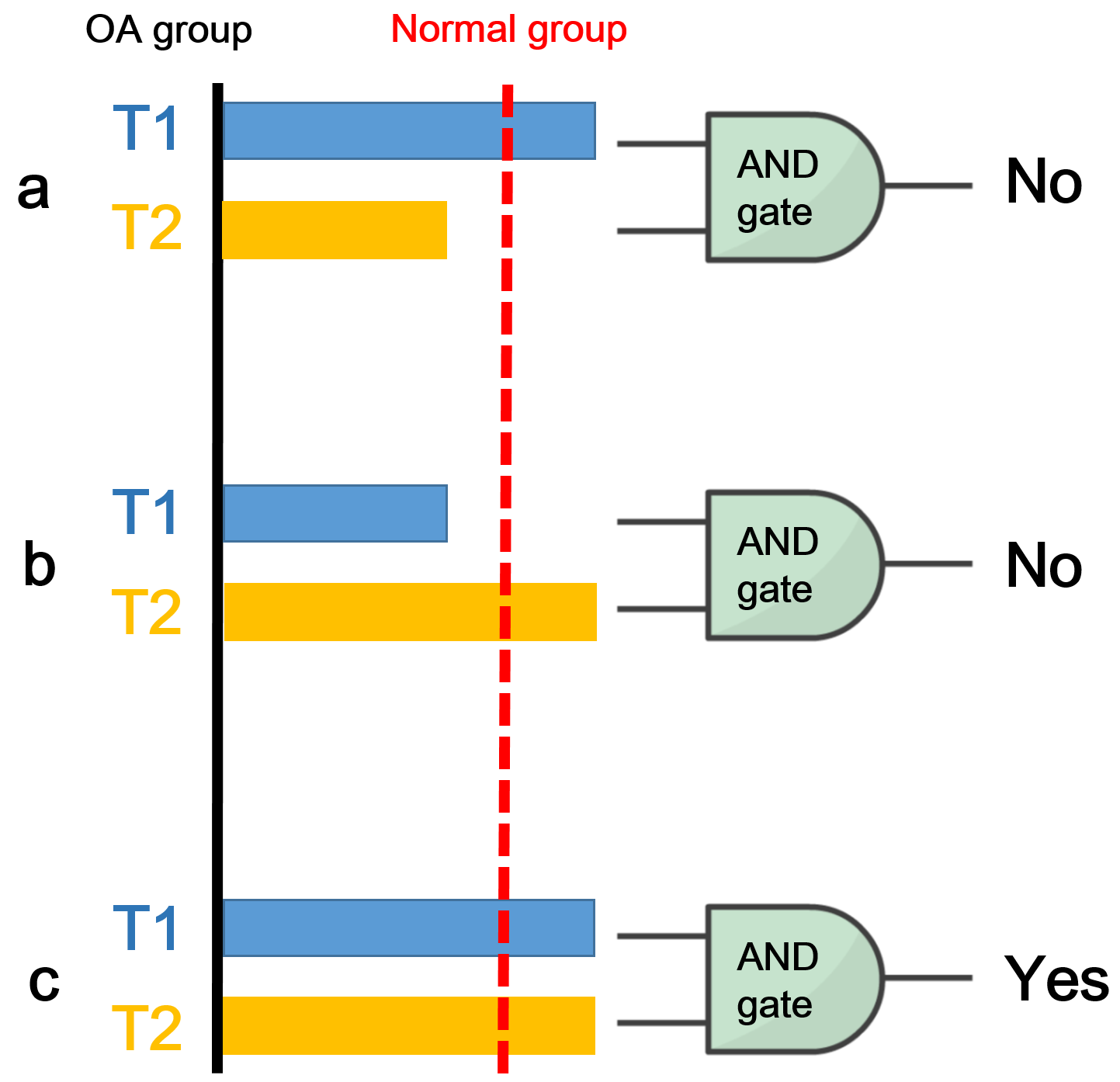


**Figure S2.** Illustration the AND logic gate process in MRI. (a, b) Ultra-small SPIONs only provide high contrast in either T1 or T2 but do not meet the criteria simultaneously for AND logic. (c) The ultra-small SPIONs in this work can provide simultaneously strong T1 and T2 value and fulfill AND logic.


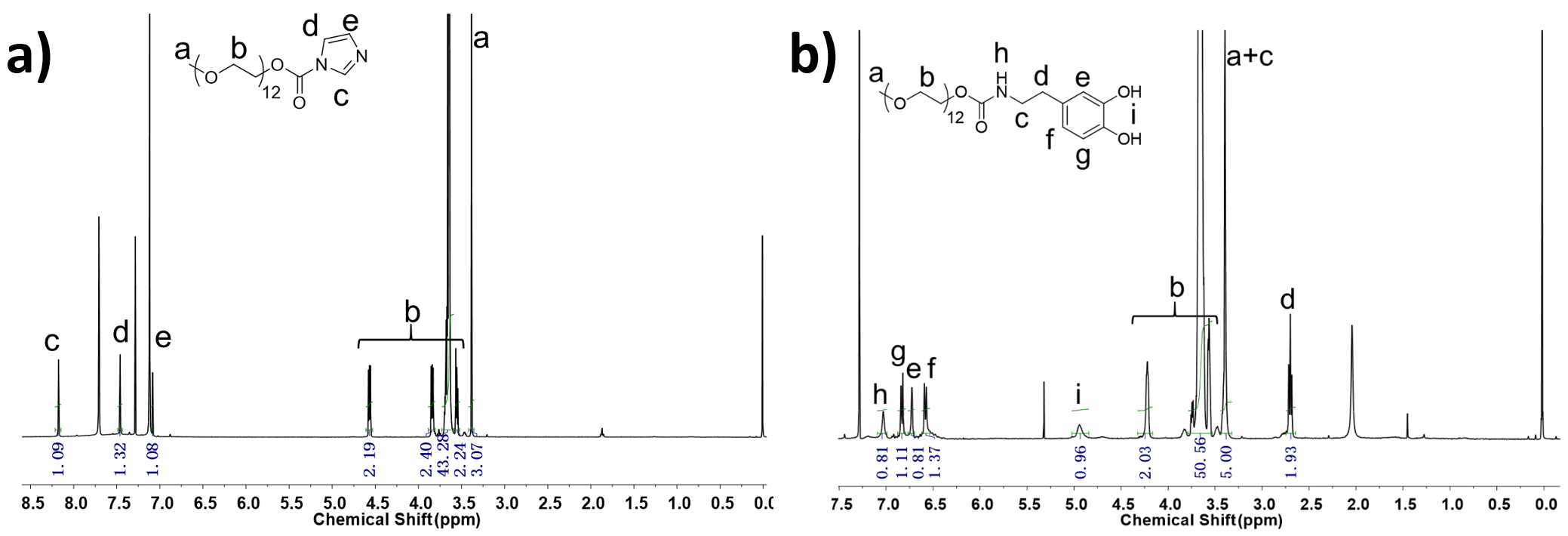


**Figure S3.**^1^H NMR spectrum (CDCl_3_) of PEG550-CI (a) and PEG550-DA (b), and the assignment of characteristic peaks.


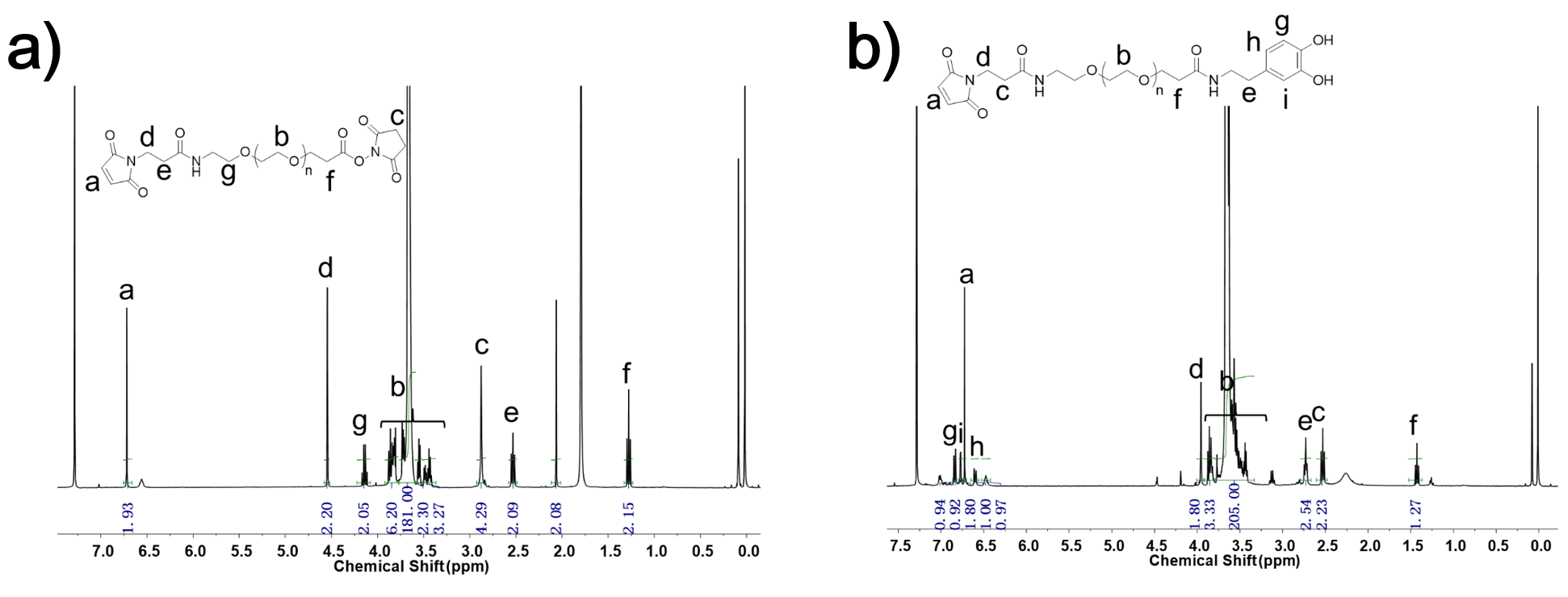


**Figure S4.**^1^H NMR spectrum (CDCl_3_) of MAL-PEG2k-SCM (a) and MAL-PEG2k-DA (b), and the assignment of characteristic peaks.


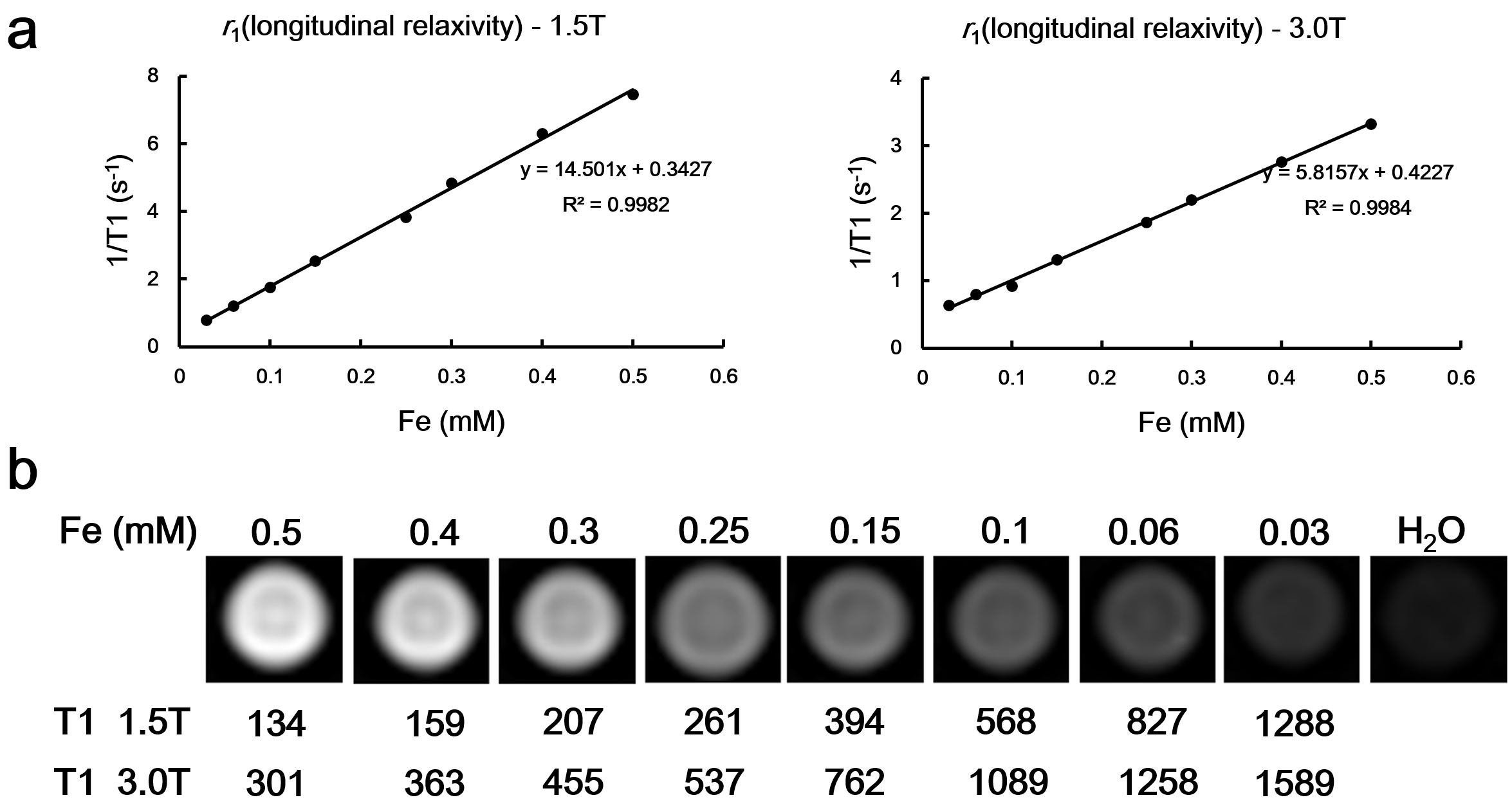


**Figure S5.** (a) The longitudinal relaxation rate (1/T1) of ultra-small SPIO nanoparticles at different iron concentrations was fitted to a curve with Fe concentration, the longitudinal relaxivity (*r*_1_) represented by the slope were 14.5 Fe mM^-1^s^-1^and 5.8 Fe mM^-1^s^-1^at 1.5T and 3.0 T, respectively. (b) T1-weighted images of ultra-small SPIONs and T1value at 1.5T and 3.0T.


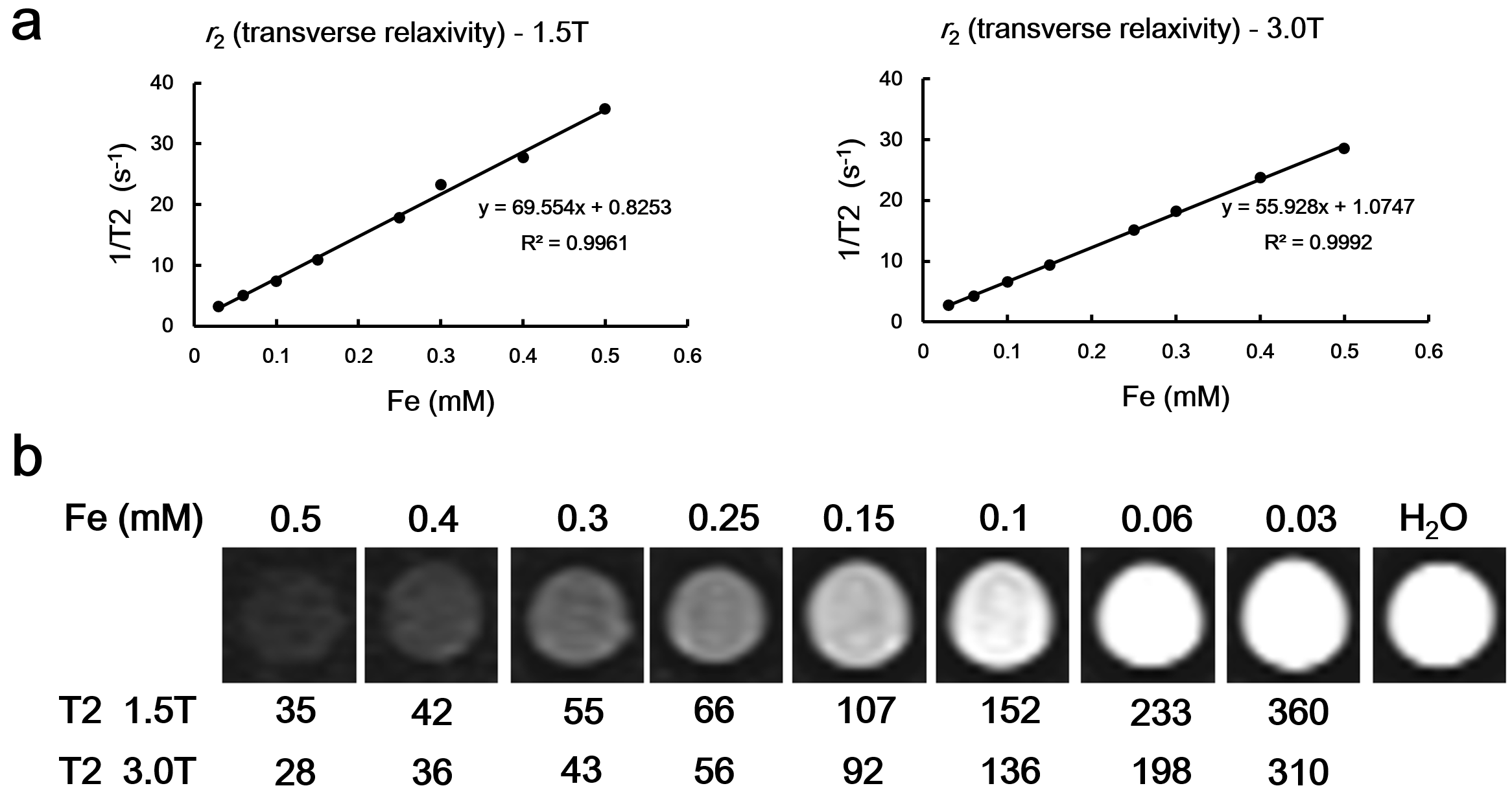


**Figure S6. (**a) The transverse relaxation rate (1/T2) of ultra-small SPIO nanoparticles at different iron concentrations was fitted to a curve with Fe concentration, the transverse relaxivity (*r*_2_) represented by the slope were 69.6 Fe mM^-1^s^-1^ and 55.9 Fe mM^-1^s^-1^at 1.5T and 3.0 T, respectively. (b) T2-weighted images of USPIONs and T1value at 1.5T and 3.0T.


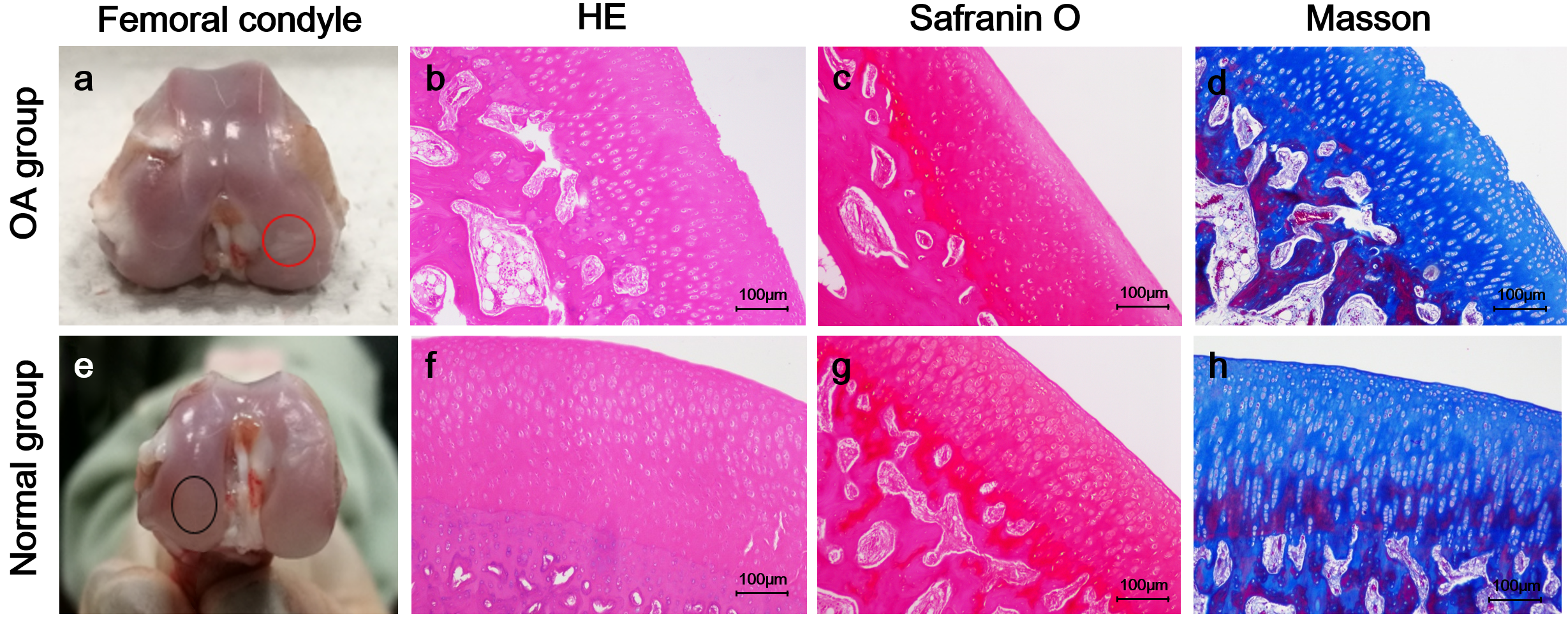


**Figure S7.** (a-d) OA group; (e-h) Normal group; (b, f) HE staining; (c, g) Safranin O staining; (d, h) Masson staining.

Table S1. T1, T2 and post/pre ratio values of articular cartilage**.**

| Time injection | Normal group (n=3) | | | | OA group (n=3) | | | |
| --- | --- | --- | --- | --- | --- | --- | --- | --- |
|  | **Targeted**  **SPIO@PEG-peptide** | | **Non-Targeted SPIO@PEG** | | **Targeted**  **SPIO@PEG-peptide** | | **Non-Targeted SPIO@PEG** | |
|  | **T1 (ms)** | **T2 (ms)** | **T1 (ms)** | **T2 (ms)** | **T1 (ms)** | **T2 (ms)** | **T1 (ms)** | **T2 (ms)** |
| Pre | **834±153** | **44±10** | **819±168** | **46±9** | **879±148** | **52±10** | **861±172** | **52±10** |
| Post 24 h | **657±150** | **30±12** | **755±149** | **35±12** | **740±154** | **38±10** | **774±132** | **43±15** |
| Post 24 h /Pre | **0.79** | **0.68** | **0.92** | **0.76** | **0.84** | **0.73** | **0.90** | **0.83** |
| Post 48 h | **674±148** | **32±9** | **757±173** | **34±9** | **780±138** | **40±11** | **766±154** | **40±8** |
| Post 48 h /Pre | **0.81** | **0.73** | **0.92** | **0.74** | **0.89** | **0.77** | **0.89** | **0.77** |
| Post 96 h | **665±145** | **30±10** | **745±144** | **38±10** | **817±117** | **46±9** | **805±158** | **44±10** |
| Post 96 h /Pre | **0.80** | **0.68** | **0.91** | **0.83** | **0.93** | **0.88** | **0.93** | **0.85** |

**Algorithm S1.**T1 and T2 map by the two-point and linear fitting method, and AND Logical Operational gorithm based on Matlab 2013.

**The two-point method to get T1map**

SE sequence T1 weighting function$I=M(1-e^{-TR/T1})$，setting TR = 0.8s, 1s, 1.2s, corresponding signal values (T1WI) are obtained, resulting in three equation functions:：$I_{1}=M(1-e^{-0.8/T1})$，$I_{2}=M(1-e^{-1/T1})$和$I_{3}=M(1-e^{-1.2/T1})$，By dividing these three equations pairwise, three comprehensive formulas are obtained：

$$\begin{matrix} I_{1}/I_{2}=(1-e^{-0.8/T1})/(1-e^{-1/T1}) \\ I_{2}/I_{3}=(1-e^{-1/T1})/(1-e^{-1.2/T1}) \\ I_{1}/I_{3}=(1-e^{-0.8/T1})/(1-e^{-1.2/T1}) \end{matrix}$$

For each of these three formulas, they are implicit function equations for the T1 value to be solved. Such implicit function equations can be approximated and solved using numerical calculation methods such as the least squares method. Theoretically, a unique solution can be obtained. Therefore, I refer to this method of solving T1 as the two-point method. After obtaining the T1 value from each formula, the average value is taken to obtain the required T1 relaxation time.

**linear fitting method to get T2map**

The formula for T2 weighting function is $I=Me^{-TE/T2}$, which is an exponential decay function. Taking the logarithm of both sides of the equation transforms the nonlinear fitting to linear fitting:$\ln I=\ln M+TE/T2$. Specifically, linear regression can be used to estimate T2 by finding the best linear fit between ln*I* and TE. This can be achieved using the least squares method through data analysis software or programming languages.

**The computer code used in this work**

%T1map,T2map, AND Logical opertation

clear;

clc;

%T1WI input

f1 = dicomread('E:\wujun\DICOM\01.dcm');

f2 = dicomread('E:\wujun\DICOM\02.dcm');

f3 = dicomread('E:\wujun\DICOM\03.dcm');

%T2WI inptut

f4 = dicomread('E:\wujun\DICOM\04.dcm');

f5 = dicomread('E:\wujun\DICOM\05.dcm');

f6 = dicomread('E:\wujun\DICOM\06.dcm');

f7 = dicomread('E:\wujun\DICOM\07.dcm');

f8 = dicomread('E:\wujun\DICOM\08.dcm');

%draw ROI in first image in series

imagesc(f3); colormap(gray);

ROI = roipoly;

hold on;

%set ROI with red line on original image;

contour(double(ROI),1,'r');

hold off;

%set Cell Array 'C'，length is 3，used to store all T1WI images

C = cell(1, 8);

%T1 WI

C{1} = f1; C{2} = f2; C{3} = f3;

%T2 WI

C{4} = f4; C{5} = f5; C{6} = f6; C{7} = f7; C{8} = f8;

mask = fspecial('gaussian');

for n = 1 : 8

f = C{n};

f = double(f);

%remove noise by Gaussian filter

for k = 1:3

f = imfilter(f, mask, 'replicate');

end

%display countours

figure; imagesc(f); colormap(gray);

hold on;

contour(double(ROI),1,'r');

hold off;

%set ROI as binary mask, multiply two images; pixel in ROI is 1 and others is 0

%assign values to Cell Array 'C'

C{n} = immultiply(ROI, f);

end

%close all;

%Initializing a zero matrix, used to store T1 value

nHeight = 512;

nWidth = 512;

ROI_T1 = zeros(nHeight,nWidth);

ROI_T2 = zeros(nHeight,nWidth);

%Initializing a zero vector, used to store T1 and T2 signal intensity

s = zeros(1, 3);

w = zeros(1, 5);

%%%%%%%%%%%%%%%%%%%%%%%%%%%%%

% T1map by two-points method

%%%%%%%%%%%%%%%%%%%%%%%%%%%%%

% merely calculating T1 value in ROI area, get T1map of ROI;

% SigI = K*(1-exp(-TR/T1)); TR = 0.8s, TR = 1.0s and TR = 1.2s

for i = 1: nHeight

for j = 1: nWidth

for n = 1 : 3

s(n) = double(C{n}(i, j));

end

%judging whether the signal vector is a zero or not

on_off = length(nonzeros(s));

if on_off ~= 0

%eps is 2.2e-16, as 0

s = s + (s == 0)*eps;

s = sort(s);

%evaluate T1 value

fun1 = @(t) (1-exp(-0.8/t))/(1-exp(-1/t))-s(1)/s(2); t1 = fzero(fun1, 1);

fun2 = @(t) (1-exp(-1/t))/(1-exp(-1.2/t))-s(2)/s(3); t2 = fzero(fun2, 1);

fun3 = @(t) (1-exp(-0.8/t))/(1-exp(-1.2/t))-s(1)/s(3); t3 = fzero(fun3, 1);

ROI_T1(i, j) = 1000*(t1+t2+t3)/ 3;

end

end

end

ROI1 = uint16(ROI_T1);

for k = 1 : 3

ROI1 = imfilter(ROI1, mask, 'replicate');

end

%binary mask, value just 0 and 1

bwROI = C{1};

%set ROI's pseudo color over grayscale image

baseImage = dicomread('E:\wujun\DICOM\03.dcm');

%data style must be 'double' rather than 'uint16'

Max = double(max(baseImage(:)));

%normalize base (anatomical) image;

baseImage = double(baseImage) / Max;

%converting to RGB (ignore colormaps)

rgbSlice = baseImage(:,:,[1 1 1]);

%show parametric image

imshow(ROI1, []);

%apply colormap

colormap('jet');

hold on;

%superimpose anatomical image

h = imshow(rgbSlice);

%make pixels in the bwROI(value = 1) display, areas of 0 value indicating transparent

set(h, 'AlphaData', ~bwROI);

colorbar('FontSize',9,'FontWeight','bold');

clim([0,1200]);

%%%%%%%%%%%%%%%%%%%%%%%%%%%%%

%T2map by the linear method

%%%%%%%%%%%%%%%%%%%%%%%%%%%%%

%merely calculating T2 value in ROI area, get T2map of ROI;

TE = [13 25 38 50 63];

%SigI = K*exp(-TE/T2);

for i = 1: nHeight

for j = 1: nWidth

for n = 1 : 5

w(n) = C{n+3}(i, j);

end

%judging whether the signal vector is a zero or not

on_off = length(nonzeros(w));

if on_off ~= 0

%eps is 2.2e-16, as 0

w = w + (w == 0)*eps;

%take the Log of signal intensity

w = log(w);

%establishing the linear regression equation: Log(SigI) = -TE/T2 + lnK

p = polyfit(TE, w, 1);

ROI_T2(i, j) = -1/p(1);

end

end

end

ROI2 = uint8(ROI_T2);

for k = 1 : 3

ROI2 = imfilter(ROI2, mask, 'replicate');

end

%binary mask, value just 0 and 1

bwROI = C{4};

%set ROI's pseudo color over grayscale image

baseImage = dicomread('E:\wujun\DICOM\04.dcm');

%data style must be 'double' rather than 'uint16'

Max = double(max(baseImage(:)));

%normalize base (anatomical) image;

baseImage = double(baseImage) / Max;

%converting to RGB (ignore colormaps)

rgbSlice = baseImage(:,:,[1 1 1]);

%show parametric image

figure, imshow(ROI2, []);

%apply colormap

colormap('jet');

hold on;

%superimpose anatomical image

h = imshow(rgbSlice);

%make pixels in the bwROI(value = 1) display, areas of 0 value indicating transparent

set(h, 'AlphaData', ~bwROI);

colorbar('FontSize',9,'FontWeight','bold');

clim([0,100]);

%background is white

set(gcf,'Color',[1 1 1]);

%%%%%%%%%%%%%%%%%%%%%%%

% T1 AND T2 logic gate

%%%%%%%%%%%%%%%%%%%%%%%

%threshhold be set by the T1 and T2 mean of Normal group in post 96h

threshold_T1 = 665;

threshold_T2 = 30;

ROI = zeros(nHeight,nWidth);

for i = 1 : nHeight

for j = 1 : nWidth

% AND logic gate

if ROI1(i, j) > threshold_T1 & ROI2(i, j) > threshold_T2

ROI(i, j) = 1;

end

end

end

figure, imshow(ROI);

%apply colormap

colormap('jet');

colorbar('FontSize',9,'FontWeight','bold');

hold on;

%superimpose anatomical image

h = imshow(rgbSlice);

%make pixels in the bwROI(value = 1) display, areas of 0 value indicating transparent

set(h, 'AlphaData', ~bwROI);

title('T1 AND T2 logic gate mapping');
